# Curative versus palliative treatments for colorectal cancer with peritoneal carcinomatosis: a systematic review and meta-analysis

**DOI:** 10.18632/oncotarget.21912

**Published:** 2017-10-20

**Authors:** Wenqiong Wu, Shipeng Yan, Xianzhen Liao, Haifang Xiao, Zhongxi Fu, Lizhang Chen, Jinsong Mou, Haibo Yu, Lian Zhao, Xiangguo Liu

**Affiliations:** ^1^ Department of Radiation Oncology, Hunan Cancer Hospital–The Affiliated Cancer Hospital of Xiangya School of Medicine, Central South University, Changsha, Hunan Province, China; ^2^ Department of Cancer Prevention and Control, Hunan Cancer Hospital–The Affiliated Cancer Hospital of Xiangya School of Medicine, Central South University, Changsha, Hunan Province, China; ^3^ Department of Chronic Diseases Prevention and Control, Centers for Disease Control and Prevention of Hunan, Changsha, Hunan Province, China; ^4^ Department of Epidemiology and Health Statistics, School of Public Health, Central South University, Changsha, Hunan Province, China; ^5^ Department of Epidemiology and Health Statistics, Changsha Medical University, Changsha, Hunan Province, China; ^6^ Department of Metabolism and Endocrinology, The Second Xiangya Hospital, Central South University, Changsha, Hunan Province, China; ^7^ Department of Gastroenterology, The Third Xiangya Hospital, Central South University, Changsha, Hunan Province, China; ^8^ Hunan Key Laboratory of Nonresolving Inflammation and Cancer, Changsha, Hunan Province, China

**Keywords:** meta-analysis, cytoreductive surgery, intraperitoneal chemotherapy, colorectal cancer, peritoneal carcinomatosis

## Abstract

The objective of this study was to provide an up-to-date summary of the current evidence that may be useful for updating guidelines. We comprehensively searched the published literatures and conferences for studies that compared curative with palliative treatments in colorectal cancer patients with peritoneal metastasis. The primary outcomes considered in this study were three- and five-year overall survival rates. We pooled data across studies and estimated summary effect sizes. Overall, patients who received curative treatments had improved three-year survival (hazard ratio (HR), 2.19 [95% CI, 1.83 to 2.62]) and five-year survival (HR, 2.22 [95% CI, 1.83 to 2.69]) compared with those who received palliative treatments. Patients who received curative treatments had an increased risk of treatment-related morbidity (odds ratio (OR), 2.90 [95% CI, 2.02 to 4.17]), but there was no significant difference in treatment-related mortality between patients who received curative treatments and those who received palliative treatments (OR, 1.46 [CI, 0.62 to 3.47]). Curative treatments improved overall survival in colorectal cancer patients with peritoneal metastasis and did not increase the risk of treatment-related mortality. Curative treatments were associated with a higher risk of treatment-related morbidity. These data highlight the importance for further investigation aimed at prevention of treatment-associated morbidity.

## INTRODUCTION

Colorectal cancer (CRC) poses an increasing threat to global health. In 2014, more than 1.4 million individuals developed CRC, and CRC-related deaths accounted for nearly 9% of the global cancer mortality burden [1]. Peritoneal carcinomatosis (PC) is a common sequela of CRC and is generally associated with limited survival [2, 3, 4, 5]. Epidemiological data indicate that approximately 5–10% of CRC patients have synchronous PC at the time of initial diagnosis, and up to 20–50% of patients with recurrent CRC will experience metachronous PC [6–9]. Without treatment intervention, median survival for most of the CRC-PC patients is only approximately 5 months. Even if palliative systemic therapy is implemented, the reported median survival time still only ranges between 5 and 15 months, which are significantly worse compared to survival times after similar therapy for other sites of CRC metastasis [10, 11, 12, 13].

Considering the poor life expectancy of CRC-PC, identifying an optimal treatment strategy to extend survival has always been a main objective of clinical oncologists. Traditionally, PC has been regarded as a terminal disease that is only amenable to palliation by systemic chemotherapy, palliative surgery or supportive care. However, with increasing knowledge regarding the patterns of CRC metastatic dissemination, experts are beginning to accept the viewpoint that PC may only be a local-regional disease entity that can be addressed in a more aggressive manner [14–16]. In this context, curative treatments such as cytoreductive surgery (CRS) in combination with intra-peritoneal chemotherapy (IPC) have emerged as promising treatments for PC [17–20].

CRS combined with IPC (CRS/IPC) is aimed at removing all visible peritoneal tumor implants by surgery firstly, followed by IPC to eliminate superficial peritoneal tumor remnants or solitary tumor cells [21, 22, 23]. Although the use of CRS/IPC has increased in recent years, it is not yet accepted as standard therapy for CRC-PC. For example, the current NCCN guidelines and Quebec guidelines still consider CRS/IPC to be experimental [24]. However, recommendations from other guidelines suggest CRS/IPC as a treatment option for selected patients [25–27]. Since there is discord among current guidelines and various experts’ opinions, further studies including one multicenter phase III trial have emerged [20]. To provide an up-to-date summary of the current evidence that may be useful in updating oncology guidelines, we conducted this meta-analysis of comparative studies that evaluated the efficacy and safety of curative treatments versus palliative treatments for CRC-PC.

## RESULTS

### Study Characteristics

**Figure 1:**
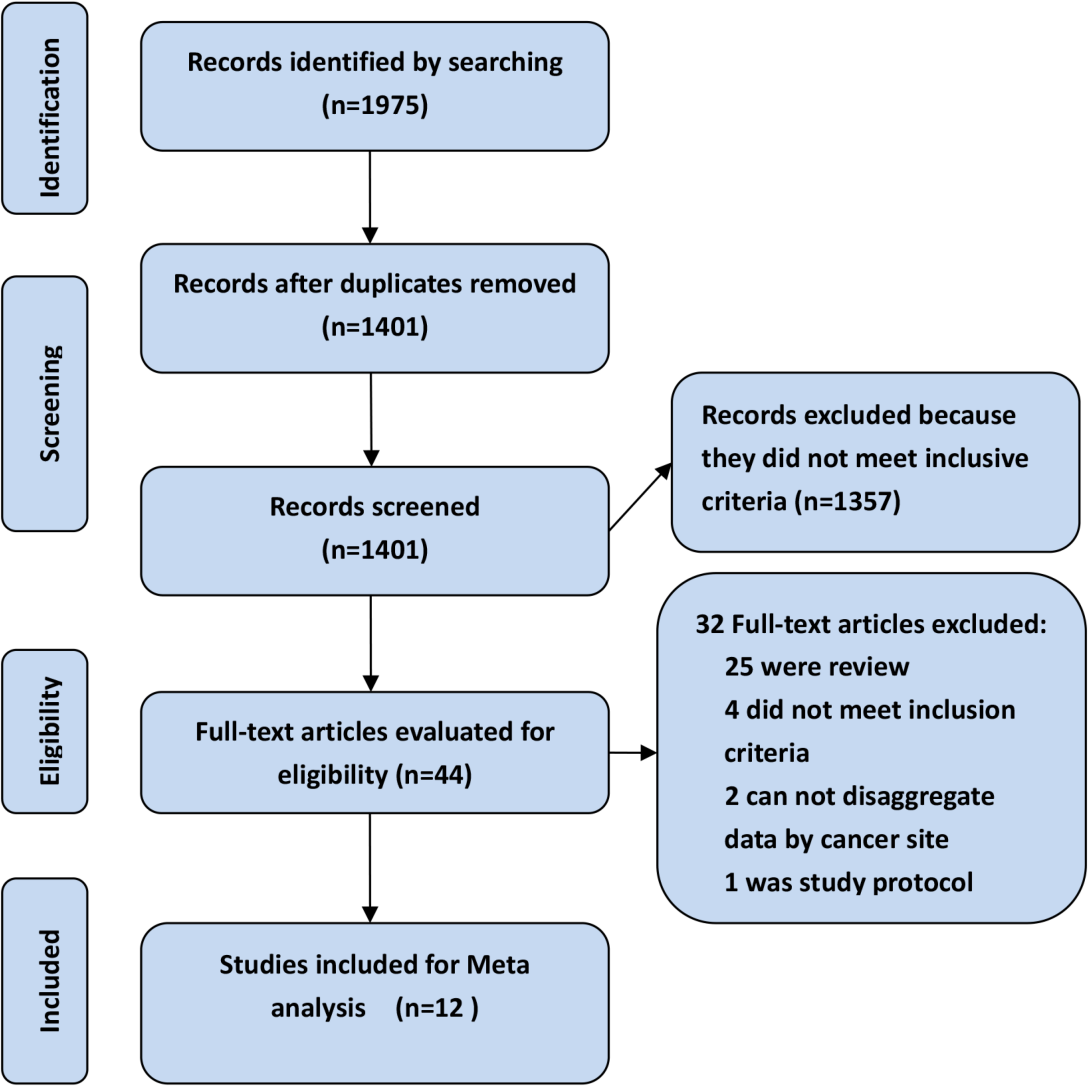
Summary of evidence search and selection

Figure 1 demonstrates the details of study identification and selection. The literature retrieval identified a total of 1975 citations. After review of the titles and abstracts, forty-four full-text articles were selected for further critical reading. Twelve articles which involved a total of 2390 patients ultimately met our inclusion criteria. All the included studies were conducted in high-volume hospitals in Asia [28, 29], Europe [20, 29, 30–36], Australia [36, 37], and North America [36, 37, 38] between 1985 and 2014 and published between 2004 and 2016. Three studies were designed as randomized controlled trial (RCT) [20, 30, 31] and the remaining nine were observational studies. The median (or mean) age of the patients ranged from 46 years [33] to 72 years [35] and the percentage of male patients varied from 27.1% [33] to 55.2% [32]. Eleven studies compared CRS/IPC to CRS (or palliative surgery) with systemic chemotherapy, and one study compared CRS/IPC with a combination of various palliative treatments [37]. The type of IPC utilized was reported by ten studies and a closed-abdomen technique was used in the most of the included studies [20, 28–30, 32, 37]. Chemotherapy protocols for IPC and systemic chemotherapy were varied, and mainly involved MMC-based, 5-FU-based and OX-based protocols. The temperature and time for the hyperthermic intra-peritoneal chemotherapy (HIPEC) procedure, reported by six studies, ranged from 40–43°C and 30–100 minutes. A summary of these studies is provided in Table 1.

**Table 1:** Characteristics of the included studies

Study name	Study design	Study location	Study period	The key Inclusions criteria of patients	curative vs. palliative treatments	Sample size	Male,%	Mean (median) age, y	Technique
Mahteme, 2004 (30)	Case-control	Sweden	1991~1999	CRC-PC ^a^	EPIC +CRS+SC vs. SC +PS	36 (18 vs. 18)	50.0	54 vs.56	CA
Elias, 2004 (31)	RCT	France	1996~2000	CRC-PC ^b^	EPIC+CRS+SC vs. SC+CRS	35 (16 vs. 19)	NA	NA	CA
Verwaal, 2008 (32)	RCT	Netherlands	1998~2007	CRC-PC ^a^	HIPEC + CRS+SC vs. SC +PS	105 (54 vs. 51)	55.2	53.0 vs.55.0	OA
Elias, 2008 (33)	Case–control	France	1998~2003	CRC-PC ^b^	HIPEC+CRS+SC vs. SC±PS	96 (48 vs. 48)	27.1	46.0 vs.51.0	CA
Franko, 2010 (38)	Case–control	USA	2001~2007	CRC-PC ^b^	HIPEC+CRS+SC vs. SC +PS	105 (67 vs. 38)	NA	51.0 vs.59.0	CA
Chua, 2011 (36)	Cohort study	USA, Australia, Germany	1988~2009	CRC-PC ^b^	EPIC+CRS vs. palliative treatments	294 (110 vs. 184)	50.0	NA	OA
Huang, 2014 (28)	Case–control	China	2004~2011	CRC-PC ^a^	HIPEC+ CRS+SC vs. SC ±CRS/PS	62 (33 vs. 29)	46.8	47.0 vs.53.0	OA
Esquivel, 2014 (37)	Cohort study	North America, Europe, Australia	1985~2012	CRC-PC^c^	HIPEC+CRS vs. SC	1013 (705 vs. 308)	52.0	57.0 vs.61.0	NA
Diane, 2015 (34)	Cohort study	France	2000~2010	CRC-PC ^b^	HIPEC/EPIC+CRS +SC vs.SC +PS	180 (139 vs. 41)	41.7	49.0 vs. 51.0	NA
Park, 2016 (29)	Case–control	Korea	2000~2013	CRC-PC ^a^	EPIC+CRS+SC vs. SC+CRS	45 (30 vs. 15)	53.3	53.5 vs. 56.0	CA
Cashin, 2016 (20)	RCT	Sweden	2004–2011	CRC-PC ^a^	EPIC+CRS vs.SC	48 (24 vs. 24)	50.0	62.0 vs. 58.0	CA
Simkens, 2016 (35)	Cohort study	Netherlands	2011~2014	CRC-PC ^b^	HIPEC+CRS+SC vs. PS+SC	371 (43 vs. 328)	53.1	66.2 vs. 71.9	OA

**Table:** 

Study name	Chemotherapy protocol for IPC	Chemotherapy protocol for SC	OS (%)		Reported median survival (months)	Incidence of treatment-related morbidity (%)	Incidence of treatment-related mortality (%)
			3-year	5-year			
Mahteme, 2004 (30)	5-FU 550 mg m-2day-1 i.p. and LV 60 mg m-2 day-1 i.v. Chemotherapy was started the day after surgery and was given daily for 6 days and repeated monthly for eight courses	5-FU+LV, MET+ 5-FU +LV	33.3 vs. 5.0	28.0 vs. 5.0	32.0 vs. 14.0	72.2 (13/18) vs.0.0 (0/18)	0.0 (0/18) vs.0.0 (0/18)
Elias, 2004 (31)	MMC on postoperative day 1 and 5-FU on postoperative days 2–5 given in a 2L solution during 23 h/24	5-FU+LV bimonthly for 6 months	39.0 vs. 44.0	NA	NA	50.0 (8/16) vs.36.8 (7/19)	18.8 (3/16) vs0.0 (0/19)
Verwaal, 2008 (32)	MMC(maximum 70 mg/m^2)^at 40°C for 90 min	5-FU (400 mg /m^2^) +LV (80 mg/ m^2^) weekly for 26 weeks or until progression or unacceptable toxicity	28.0 vs. 19.0	19.0 vs. 10.0	22.3 vs. 12.6	NA	7.5 (4/53) vs.0.0 (0/55)
Elias, 2008 (33)	OX (460mg /m^2^) at 42°C for 30min. Before HIPEC, patients received 5-FU 400 mg/m^2^ + LV20 mg/m^2^ i.v.	5-FU based (46/48), CAP-based(1/48), CAM-based(1/48)	72.0 vs. 31.0	51.0 vs. 13.0	62.7 vs. 23.9	NA	NA
Franko, 2010 (38)	MMC 40 mg at 42°C for 100 min	5-FU+IRI, OX+BEV and /or CET	49.0 vs. 20.0	27.0 vs. 8.0	34.7 vs. 16.8	NA	NA
Chua, 2011 (36)	MMC(10–12.5 mg/m^2^) at 42°C for 90 min	5-FU+LV(43/294),CAP+OX/IRI (105/294),CAP+ BEV/CET/PAN(76/294)	55.0 vs. 15.0	30.0 vs. 5.0	NA	NA	NA
Huang, 2014 (28)	CIS (120mg)+MMC (30mg) at 43± 0.5°C for 90min	Ox+LV+5-FU , IRI+LV+5-FU	16.0 vs. 0.0	NA	13.7 vs. 8.5	28.6 (10/35) vs.9.4 (3/32)	0.0 (0/35) vs.6.3 (2/32)
Esquivel, 2014 (37)	OX only (166/705), MMC only (354/705), Others (67/705), data missing (118/705)	NA	66.0 vs25.0	58.0 vs. 19.0	41.0 vs. 10.0	NA	NA
Diane, 2015 (34)	HIPEC (121/139): OX ± IRI (87/121), OX alone (34/121) EPIC (18/139):MMC+5-FU	5-FU +OX (105/180) , 5-FU+IRI (83/180), 5-FU alone (1/180), BEV(49/180), CET(13/180)	52.0 vs. 7.0	30.0 vs. 0.0	NA	52.5 (73/139) vs.19.5 (8/41)	5.8 (8/139) vs.4.9 (2/41)
Park, 2016 (29)	MMC (10 mg/m^2^/d)+ 5-FU (700 mg/m^2^/d)	OX ± IRI based mainly	74.3 vs. 34.7	65.0 vs. 23.0	NA	23.3 (7/30) vs.26.7 (4/15)	3.3 (1/30) vs.0.0 (0/15)
Cashin, 2016 (20)	5-FU (550 mg /m^2^/d)i.p. + LV (30 mg /m^2^/d) i.v.	5-FU+ LV+OX or IRI	37.5 vs. 20.8	33.0 vs. 4.0	25.0 vs. 18.0	41.7 (10/24) vs.50.0 (12/24)	0.0 (0/24) vs.0.0 (0/24)
Simkens, 2016 (35)	MM C (35 mg/m^2^) at 41°C for 90 min or OX (460 mg mg/m^2^) at 41°C for 30 min	NA	NA	NA	NA	69.8 (30/43) vs.40.5 (133/328)	0.0 (0/43) vs.3.4 (11/328)

Abbreviations: CRS = cytoreductive surgery; PS = palliative surgery; IPC = intraperitoneal chemotherapy; HIPEC = hyperthermicintraperitoneal chemotherapy; EPIC = early postoperative intraperitoneal chemotherapy; CA = closed abdomen; OA = opened abdomen; NA = not available; OS = overall survival; SC = systemic chemotherapy; 5-FU = 5-fluorouracil; LV = leucovorin; MET = methotrexate; MMC = mytomycin C; OX = oxaliplatin; CAP = capecitabine; CAM = camptothecin; IRI = irinotecan; CIS = cisplatin; BEV = bevacizumab; CET = cetuximab; PAN = panitumumab;

NOTE: 1: ^a^Patients without any extra-peritoneal metastasis; bPatients with extra-peritoneal metastasis (included liver, lymph or other extra-abdominal metastasis);^c^ not clearly described; 2: If not specified, data were arranged by curative vs. palliative.

### Quality assessment of included studies

**Figure 2:**
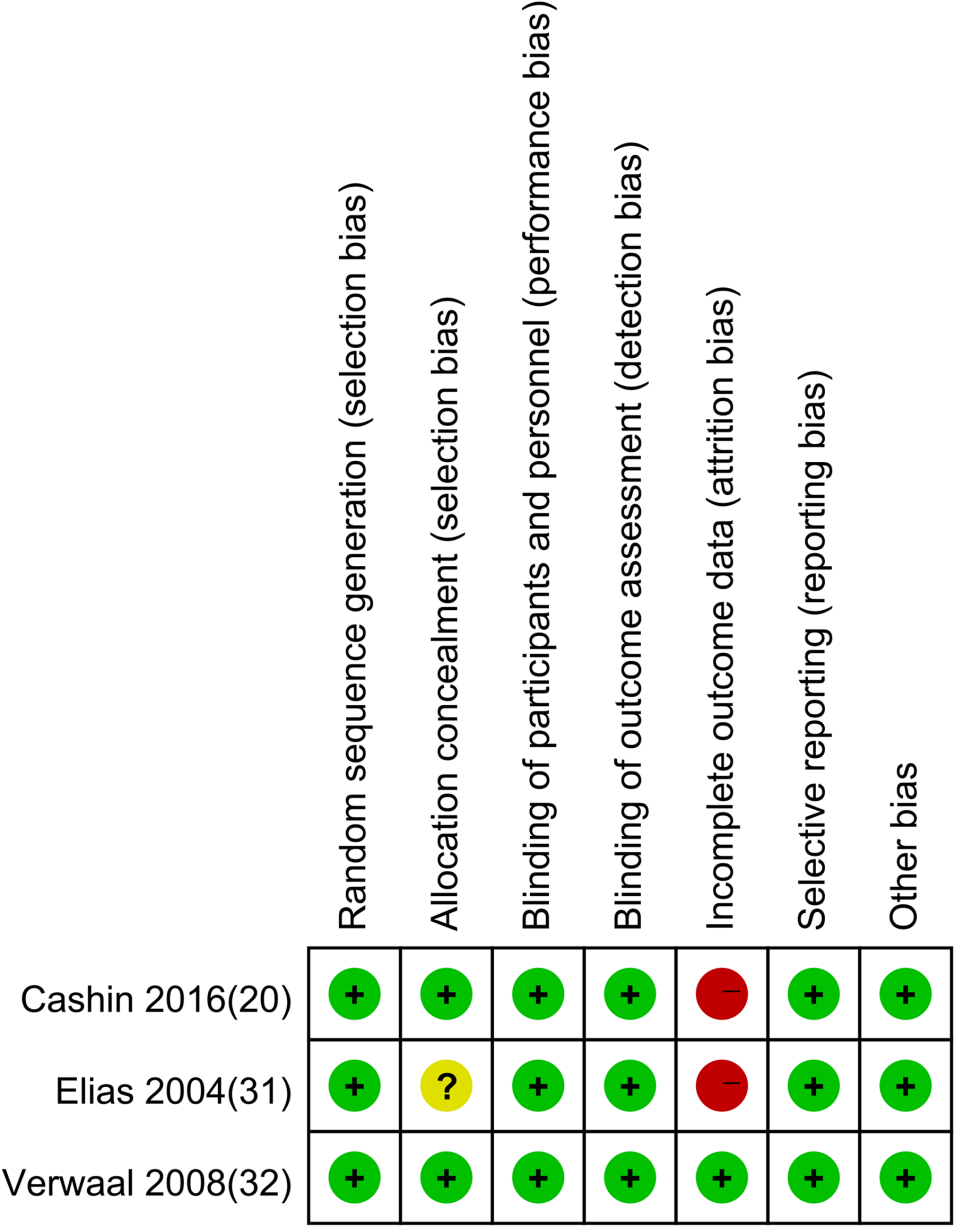
Risk of bias assessment of included RCTs

The risk of bias evaluation of the three included RCTs is displayed in Figure 2. Allocation sequence generation was appropriate in all trials and allocation concealment was appropriate in two trials but vague in the one remaining trial [31]. All of the included trials were open-label trials in which patients, researchers and clinical personnel were not blinded to treatment allocation. One trial [20] clearly stated that they did not mask the outcome evaluator to treatment allocation, but it was unclear in the remaining two trials. In two trials [20, 30], the studies were ended before reaching the required number of patients, which may have increased the potential risk of bias due to incomplete data. No evidence of selective outcome reporting or obvious other sources of bias were detected. In nine observational studies, the methodological quality assessment yielded an average score of 7.8, and 77.8% of the studies were very high quality (Supplementary Tables 1 and 2). Nevertheless, because of the inherent limitations of randomization, baseline imbalance can be found in the majority of the included studies [33–38].

### Overall survival

**Figure 3:**
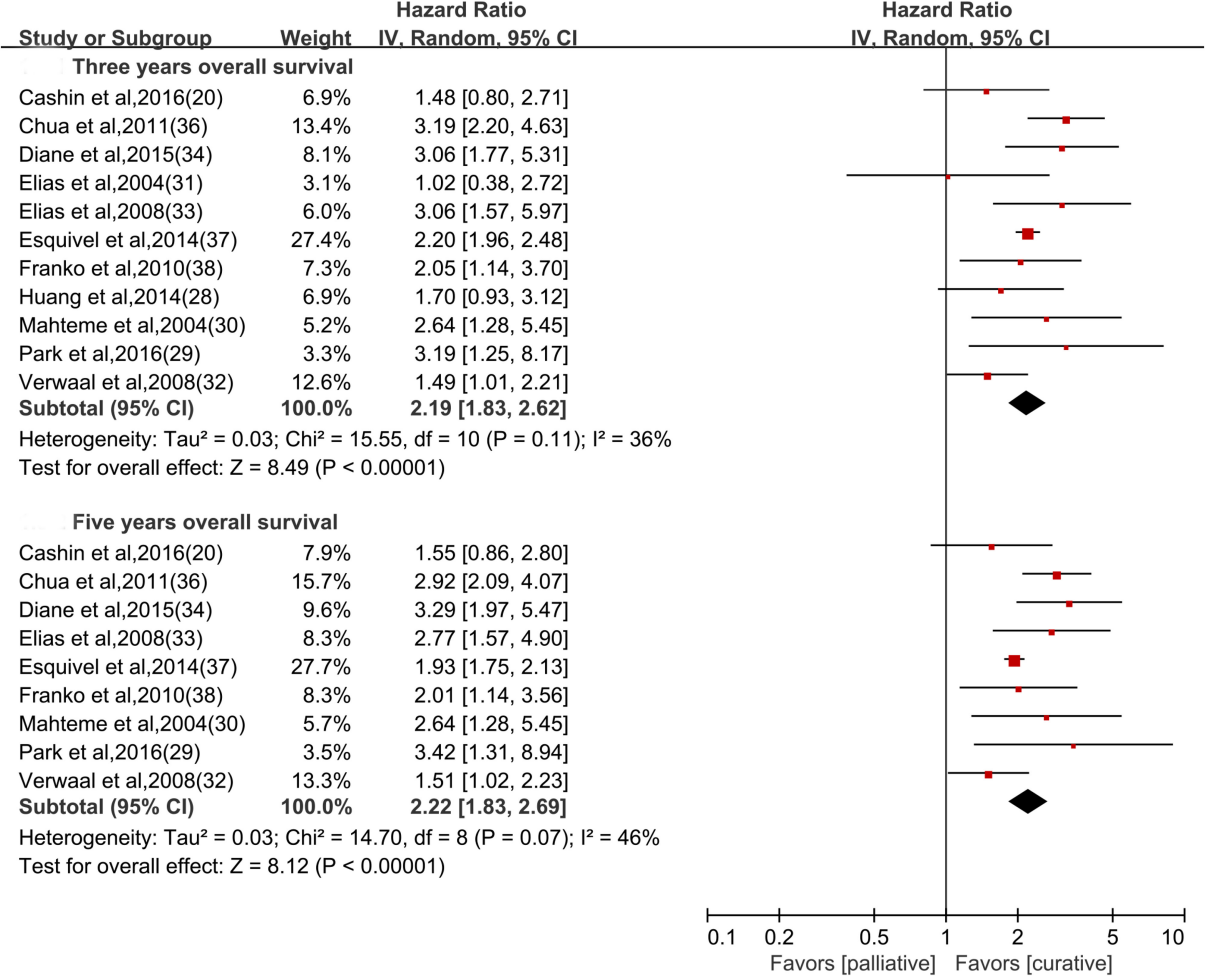
Overall survival comparing curative versus palliative treatments, stratified by three- and five-year survival

The hazard ratios (HRs) of survival data in all included studies were calculated based on the information extracted from Kaplan-Meier survival plots. Overall, patients who received curative treatments (53.8% [1287/2390 participants]) had improved short-term and long-term overall survival compared to those who received palliative treatments (46.2% [1103/2390 participants]) at the end of follow up. The pooled HR was 2.19 for three-year survival (11 studies; [95% CI 1.83~2.62], I^2^ = 36.0%), and 2.22 for five-year survival (9 studies; [95% CI 1.83~2.69], I^2^ = 46.0%) (Figure 3). In a pre-specified subgroup analysis, the survival benefit among patients who received curative treatments was less susceptible to the criteria of enrollment, study design, IPC type, IPC technique and IPC chemotherapy protocol (Supplementary Table 3).

### Overall treatment-associated morbidity and mortality

**Figure 4:**
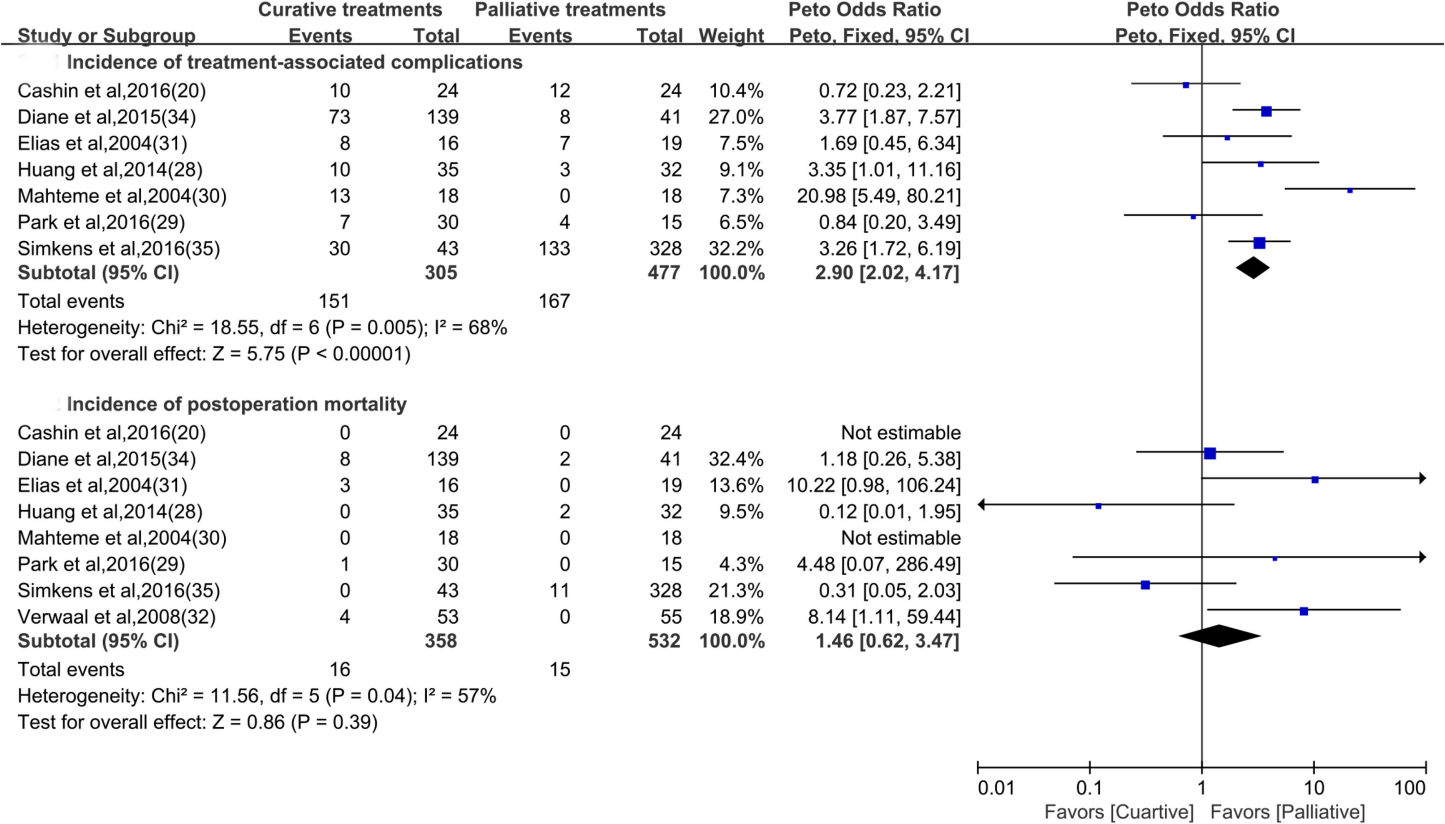
Overall treatment-associated morbidity and mortality comparing curative versus palliative treatments

Seven studies provided data on the overall treatment-associated morbidity. Among 305 patients who received curative treatments, 151 (49.5%) developed treatment-associated complications compared to 167 out of a total of 477 patients (35.0%) in the palliative treatment group. In eight studies, 31 deaths were recorded, of which 16 deaths (4.5%, 16/358) occurred in the curative treatment group and 15 deaths (2.8%, 15/532) occurred in the palliative treatments group. As shown in Figure 4, patients who received curative treatments had a significantly higher risk of treatment-associated complications (Odds Ratio (OR), 2.90 [95% CI, 2.02 to 4.17]; I^2^ = 68.0%), but there was no evidence indicating that curative treatments were associated with an increased treatment-related mortality (OR, 1.46 [CI, 0.62 to 3.47]; I^2^ = 57.0%).

### Other secondary outcomes

Individual and pooled HRs or ORs for other secondary outcomes are displayed in Supplementary Figures 1 and 2. Patients who received curative treatments had prolonged three-year disease-free survival (2 studies; HR, 1.43 [95% CI, 1.01 to 2.03]; I^2^ = 84%) and three-year peritoneal-disease-free survival (1 study; HR, 2.51 [95% CI, 1.16 to 5.44]), and had a decreased risk of recurrence (1 study; OR, 0.08 [95% CI, 0.01 to 0.70]). In the group that received curative treatments, the rate of readmission (1 study; OR, 3.87 [95% CI, 1.64 to 9.12]) was higher than in the group who received palliative treatments. No statistically significant differences between groups with respect to the rate of termination of the planned therapy were found (2 studies; OR, 1.89 [95% CI, 0.77 to 4.62]; I^2^ = 40%). Because of the statistically significant heterogeneity in the method of data analysis or an irregular type of data, some studies could not be combined for meta-analysis. Verwaal et al. [32] reported a longer median disease-free survival in patients who received CRS plus HIPEC (12.6 months vs. 7.7 months, *p* = 0.020). In addition, Park et al. [29] and Simkens et al. [35] found a significantly longer duration of hospital stay in the CRS/IPC group.

### Sensitivity analysis and publication bias

To further assess the outcomes of the primary analysis, we conducted a sensitivity analysis. After removing each study sequentially from the pooled analysis, the pooled HR only changed slightly (for three-and five-year survival, HR ranged from 2.18 to 2.38 and 2.28 to 2.53, respectively). Funnel plots and Egger's weighted regression (three-year survival: *p* = 0.80; five-year survival: *p* = 0.14) showed that there was no publication bias (Supplementary Figures 3 and 4).

## DISCUSSION

While a body of cohort reports [39–41] and phase II studies [9, 19, 42] have suggested a survival benefit for patients with CRC-PC who received curative treatments, evidence from comparative studies with internal controls alone is still not enough. In this systematic review and meta-analysis evaluating the survival and clinical outcomes of curative versus palliative treatments in patients with CRC-PC, we included 12 studies with 2761 comparable patients. To our knowledge, this is by far the largest meta-analysis on CRC-PC treatment including extensive data from comparative studies. Across all studies, we found a statistically significant improvement in short-term (three-year) and long-term (five-year) overall survival in those patients who received curative treatments, although these treatments bear a higher risk of treatment-associated complications. In addition, no evidence indicated that curative treatment was associated with increased treatment-related mortality.

Although results derived from this meta-analysis strongly support that selected patients with CRC-PC may benefit from curative treatments, they also emphasize the need for more detailed data (such as a cohort analysis or subgroup analysis of existing studies) to assess the impact of other factors on clinical treatment endpoints of these curative treatments. We noticed that quite a few factors, such as higher Peritoneal Cancer Index [28, 34], higher Peritoneal Surface Disease Severity Score [36, 37], and incomplete cytoreduction [28, 32], might moderate the positive effect of curative treatments, but we were unable to explore these factors thoroughly due to limited data. We also realize that some issues, such as the adjunctive contribution of IPC to CRS, optimal chemotherapy regimens, and standardization of the IPC procedure, still require further study. However, until such evidence becomes available to better define the threshold for universal application of curative treatments for CRC-PC, current guidelines should acknowledge this strong evidence for the use of curative treatments in selected patients with CRC-PC.

Overall survival benefits, although important, are certainly not the only concern in treating patients with CRC-PC. Treatment of CRC-PC requires high patient compliance, consideration of treatment-associated morbidity and mortality, and assessment of the risk for disease progression or recurrence. Our meta-analysis, although underpowered with regard to many of these important secondary outcomes, indicated that curative treatments could prolong disease-free survival and peritoneal-disease-free survival, decreases the risk of recurrence, and did not show any statistically significant difference in treatment-associated mortality or termination of the planned treatment. Curative treatments were associated with increased treatment-related complications, longer duration of hospital stay, and higher risk for short-term readmission. These data highlight the importance of further investigation aimed at prevention of treatment-associated morbidity in high-volume hospitals where CRS and IPC are widely implemented, by improving patient enrollment criteria, optimizing CRS and IPC procedures, and intensifying perioperative management.

Our search for systematic reviews and meta-analyses on curative versus palliative treatments in patients with CRC-PC was updated in December 2016 and 2 additional published studies were identified. Cao et al. [43] concluded that combined therapy involving CRS/IPC had a statistically significant survival benefit over control groups. Mirnezami and colleagues [44] also concluded that, in carefully selected patients, CRS plus HIPEC had a strong positive impact on prognosis for medium- and long-term survival compared with systemic chemotherapy alone. However, both reviews only included four comparative studies and the investigators failed to provide reliable estimates for some primary and secondary outcomes (such as disease-free survival, incidence of treatment-related morbidity and mortality, and the frequency of termination of the planned treatment) using meta-analysis.

Strengths of this review include the detailed search strategy used to identify all potential eligible comparative studies on the treatment of CRC-PC. Furthermore, we assessed the risk of bias rigidly and applied rigorous means to control the bias during the analytic process. There are, however, several limitations of the present study. Based on the information available to us, we considered the majority of the included studies to be high quality. However, most of the included studies were retrospective nonrandomized studies, and thus inevitably subject to potential bias as a result of unmeasured variables that could have affected outcomes, for example an imbalance in baseline characteristics between groups. Another limitation is the issue of heterogeneity. Clinical and methodological diversity can be observed in any meta-analysis. Although we conducted subgroup analysis and utilized a random-effects model to assess and control the impact of heterogeneity, clinical heterogeneity (such as different criteria of patient enrollment, IPC type and technique, and chemotherapy protocol) between studies may still be a potential source of bias in our report. Finally, although we used the endpoint of overall treatment-associated complications as reported from individual studies, we acknowledge the possibility of adjudication of related events due to lack of a uniform morbidity classification system.

## MATERIALS AND METHODS

This systematic review and meta-analysis was conducted according to PRISMA (Preferred Reporting Items for Systematic Reviews and Meta-Analyses) guidelines, and the background, rationale and basic methods utilized were specified in advance and documented in the PROSPERO database (CRD: 42016036628).

### Data sources and search strategy

We systematically searched the PubMed, Ovid, Embase, Web of Science, International Clinical Trials Registry Platform, and Cochrane Library up until December 31, 2016. We applied a highly sensitive search strategy, using a combination of the search terms “intraperitoneal chemotherapy”, “hyperthermia intraperitoneal chemotherapy”, “cytoreductive surgery”, “colorectal cancer (or colorectal carcinoma)”, and “peritoneal carcinomatosis” to conduct literature retrieval without language restriction. Furthermore, we searched for abstracts from major cancer conferences from 2016 onward, including the American Society of Clinical Oncology Conference, the American Association for Cancer Research Conference, the European Society for Medical Oncology Conference, the European Cancer Congress, the European Society for Therapeutic Radiology and Oncology Conference, the World Congress on Gastrointestinal Cancer, and the World Cancer Congress. In addition, we identified other potentially eligible studies from the reference lists of studies identified through the search methods described above.

### Study selection

Two researchers independently assessed the eligibility of studies obtained from the literature retrieval. Any disagreements were resolved through discussion, and the agreements were reached by consensus. We included studies that provided comparative data for curative treatments (CRS/IPC) versus palliative treatments (any combination of supportive care, systemic chemotherapy or palliative surgery) in adult patients with synchronous or metachronous CRC-PC. If the study reported comparative outcomes for different cancer subtypes, we only included data for patients with CRC-PC. When multiple articles reported on the same study, only the most recent or most informative publications were included. The primary outcomes considered in this study were three- and five-year overall survival rates. Secondary outcomes included incidence of treatment-related morbidity and mortality, disease-free survival, peritoneal-disease-free survival, frequency of termination of the planned treatment, recurrence rate of CRC-PC, and short-term readmission.

### Data extraction and quality assessment

Two researchers independently extracted and checked the data. For each included study, detailed information regarding the authors, year of publication, characteristics of the study population, study design, interventions, and outcome measures were extracted and recorded. If the study did not report accurate survival data and the authors did not respond to our inquiries, we extracted the data from Kaplan-Meier survival plots using graphic digitization software according to previously published methodology [45]. Any disagreements on the extracted data were resolved via consensus. The Cochrane Collaboration's risk of bias tool [46] was used to assess the risk of bias of the included RCTs from six domains, including sequence generation, allocation concealment, use of a blind method, incomplete outcome data, selective data reporting and other sources of bias. The Newcastle-Ottawa Scale star system [47] was used to evaluate the methodological quality of observational studies. A study awarded seven or more stars was regarded as a high-quality study [48]. Authors of the included studies were contacted for additional unpublished information to use in the risk of bias evaluation and planned subgroup analysis if necessary.

### Data integration and statistical analysis

In this study, results regarding the survival outcomes and other dichotomous outcomes were expressed as HR and OR respectively. For studies that did not report HR from survival analysis, HR and 95% confidence intervals (CI) were calculated from Kaplan-Meier survival curves by using a hierarchical series of steps described by Tierney [49]. HR or OR with 95% CI were pooled by a fixed effects model or random effects model according to the heterogeneity assumption. The degree of heterogeneity across studies was measured and quantified using chi-square testing and I^2^ statistics [50]. Funnel plots and Egger's test were used to evaluate the publication bias. When publication bias existed, a nonparametric trim-and-fill method was used to adjust the primary results of the meta-analysis [51]. We also conducted a sensitivity analysis by excluding studies one by one from the overall analysis to measure the stability of our primary analysis. When studies could not be combined for meta-analysis due to significantly clinical heterogeneity, narrative syntheses were conducted. The data from individual studies were presented graphically to offer a concise summary of evidence. Subgroup analysis for primary outcomes was pre-specified to assess the effects in patients with different study design, criteria of enrollment, IPC type (IPC vs. HIPEC), IPC technique (closed abdomen vs. open abdomen), and IPC chemotherapy protocol (combined chemotherapy vs. mono-chemotherapy).

EndNote (VersionX7, Thomson Corp) was used for bibliographic citation management. Stata software (Version 13.0, Stata Corp) and Review Manager (Version 5.2, Nordic Cochrane Centre) were used for the subsequent meta-analysis.

## CONCLUSIONS

Although a significant increase in the frequency of treatment-related morbidity, the evidence indicated that curative treatment improved survival in patients with CRC-PC. Curative treatment may also prolong a patient's disease-free survival and peritoneal-disease-free survival, decrease the risk of recurrence, and does not increase the risk of treatment-related mortality. In light of the existing evidence, we suggest that current clinical guidelines should be updated to support curative treatments as an alternative choice for patients with CRC-PC and call for further research to address the threshold at which the survival benefit associated with curative treatment begins to attenuate.

## SUPPLEMENTARY MATERIALS FIGURES AND TABLES


