# Supplementary material for: Curative versus palliative treatments for colorectal cancer with peritoneal carcinomatosis: a systematic review and meta-analysis
Source: Oncotarget. 2017 Oct 20;8(68):113202–12. doi: 10.18632/oncotarget.21912 (PMC5762584; doi:10.18632/oncotarget.21912)
Supplement: Supplementary file 1 [file oncotarget-08-113202-s001.pdf]

Curative versus palliative treatments for colorectal cancer with peritoneal carcinomatosis: a systematic review and meta-analysis

SUPPLEMENTARY MATERIALS

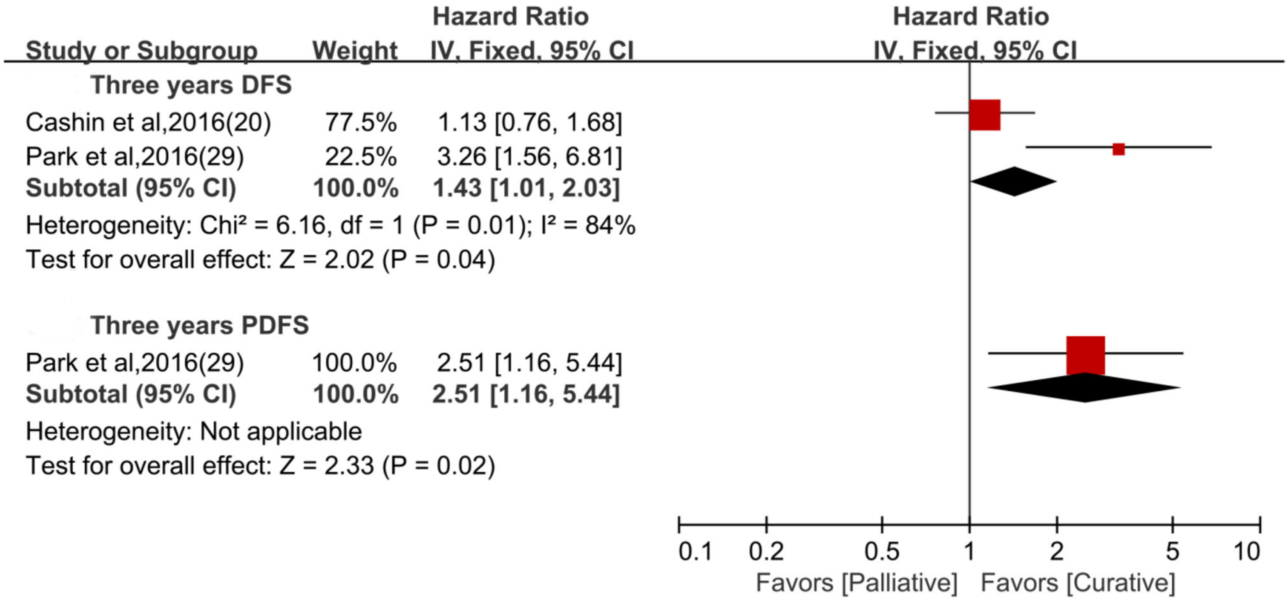

Supplementary Figure 1: Three-year DFS and P-DFS comparing curative treatments versus palliative treatments. Note: DFS= disease-free survival; P-DFS=peritoneal-disease-free survival.

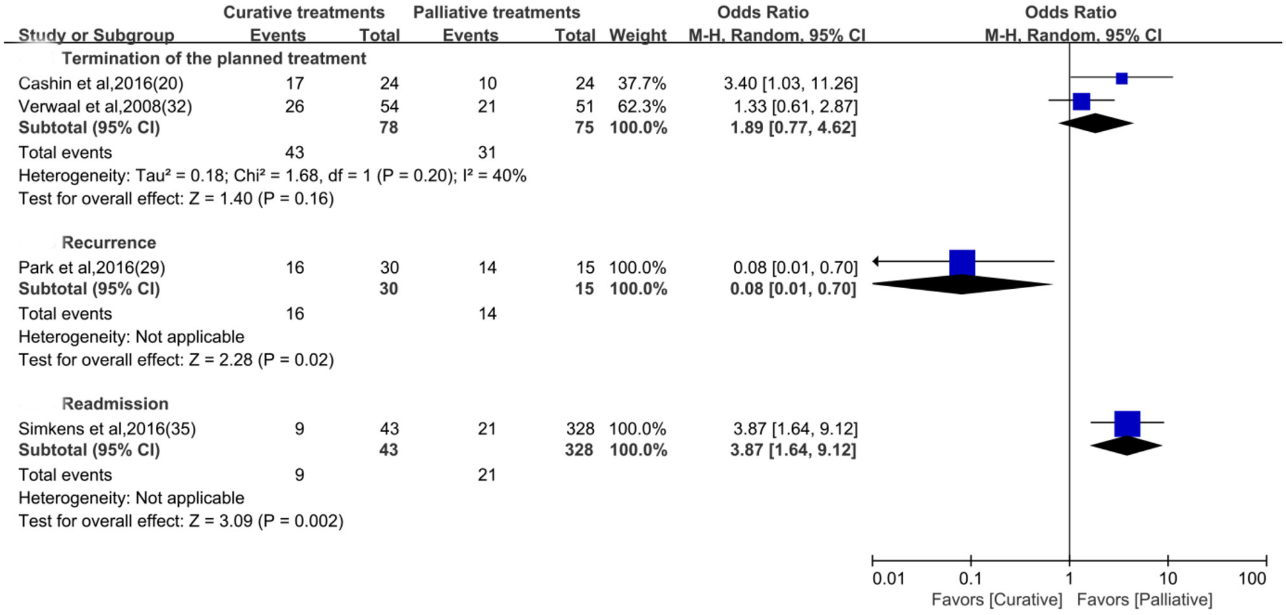

Supplementary Figure 2: Termination of the planned treatment, recurrence, and short-term readmission comparing curative treatments versus palliative treatments.

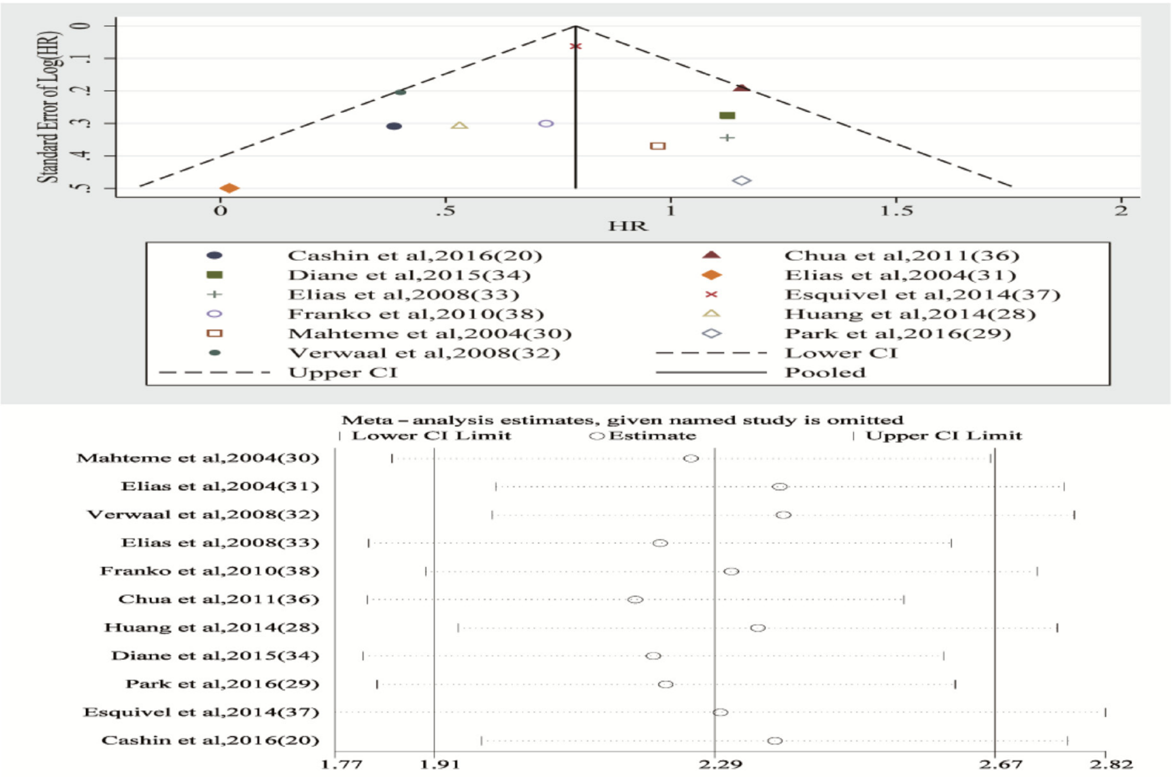

Supplementary Figure 3: Sensitive analysis and funnel plot for three-year survival.

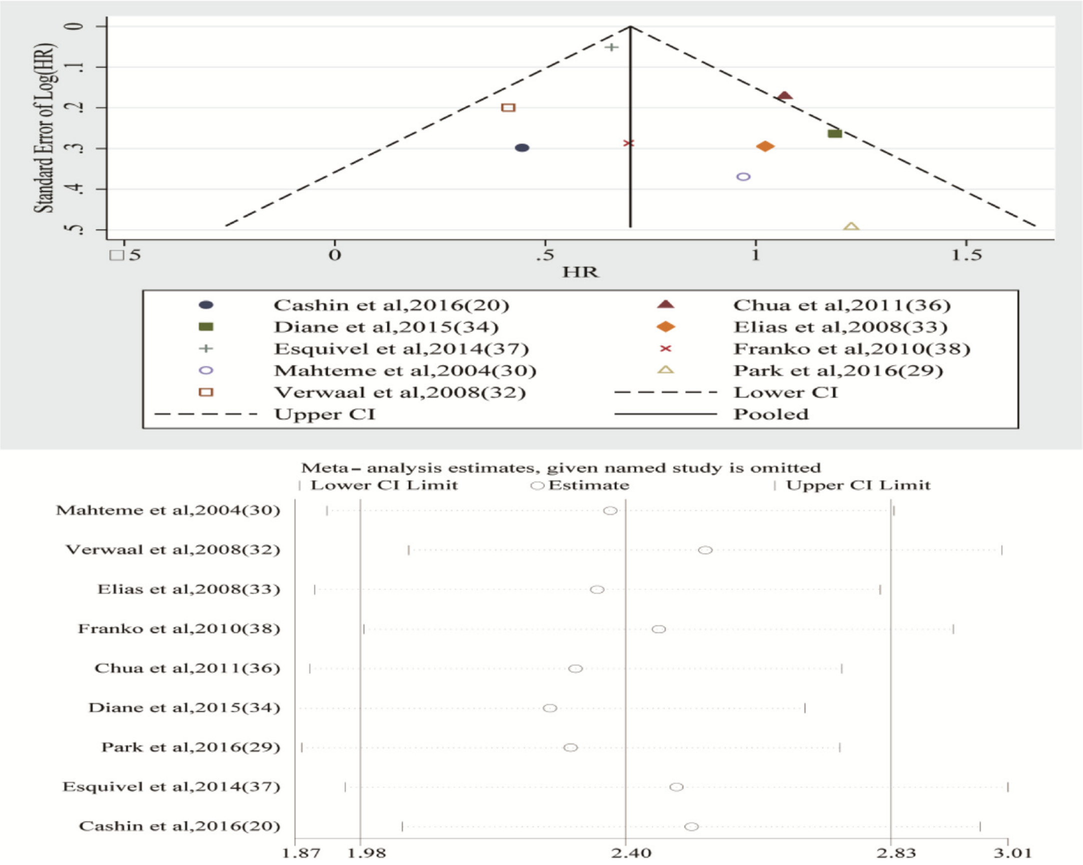

Supplementary Figure 4: Sensitive analysis and funnel plot for five-year survival.

Supplementary Table 1: Results of the methodological quality assessments for case-control study using the newcastle-ottawa scale (ONS)

Pubilications	Selection					Exposure			Total score
	Definition adequate	Representativeness of the cases	Selection of Controls	Definition of Controls	Comparability between groups	Ascertainment of exposure	Ascertainment for cases and controls	Non-Response rate	
Mahteme, 2004 (30)	*	*		*	**	*	*	*	8
Elias, 2008 (33)	*	*		*	*	*	*	*	7
Franko, 2010 (38)	*	*		*	*	*	*	*	7
Huang, 2014 (28)	*	*		*	**	*	*	*	8
Park, 2016 (29)	*	*		*	**	*	*	*	8

Supplementary Table 2: Results of the methodological quality assessments for cohort study using The Newcastle-Ottawa Scale (ONS)

Pubilications	Selection					Outcome			Total score
	Representativeness of the exposed cohort	Selection of the non-exposed cohort	Ascertainment of exposure	Outcome present	Comparability of cohorts	Assessment of outcome	follow-up long enough for outcomes	Adequacy of follow up of cohorts	
Chua,2011(36)	*	*	*	*	*	*	*	*	8
Esquivel,2014(37)	*	*	*	*	*	*	*	*	8
Diane,2015(34)	*	*	*	*	*	*	*	*	8
Simkens,2016(35)	*	*	*	*	*	*	*	*	8

Supplementary Table 3: The pooled hazard ratio for curative versus palliative treatments among CRC-PC patients, by study subgroup

Subgroup	Overall survival	No.of studies	HR	95% CI	Heterogeneity (I^2, %)
**Overall**	3-year	11	2.19	1.83~2.62	36.0
	5-year	9	2.22	1.83~2.69	46.0
**Study design**					
RCT	3-year	8	2.25	2.00~2.49	0.0
	5-year	7	2.00	1.81~2.19	18.9
Non-RCT	3-year	3	1.41	0.94~1.88	0.0
	5-year	2	1.52	1.01~2.04	0.0
**criteria of enrollment**					
CRC-PCa	3-year	5	1.60	1.15~2.05	0.0
	5-year	4	1.62	1.12~2.11	0.0
CRC-PCb	3-year	5	2.36	1.44~3.28	50.1
	5-year	4	2.68	2.03~3.32	0.0
**IPC type**					
EPIC based	3-year	5	1.89	1.30~2.48	51.1
	5-year	4	2.29	1.64~2.94	27.3
HIPEC based	3-year	5	2.08	1.84~2.31	27.6
	5-year	4	1.90	1.72~2.09	0.0
**IPC technique**					
Closed abdomen	3-year	6	1.72	1.15~2.30	0.0
	5 years	5	2.02	1.38~2.66	0.0
Open abdomen	3-year	3	1.80	1.31~2.29	25.0
	5-year	2	1.89	1.37~2.40	26.3
**IPC chemotherapy protocol**					
Combined chemotherapy	3-year	6	1.65	1.10~2.21	0.0
	5-year	4	2.02	1.26~2.79	0.0
Mono-chemotherapy	3-year	3	1.86	1.36~2.37	65.9
	5-year	3	1.91	1.43~2.38	64.2

NOTE: aPatients without any extra-peritoneal metastasis; bPatients with extra-peritoneal metastasis (included liver, lymph or other extra-abdominal metastasis).
